# Revision of the ant genus *Proceratium* Roger (Hymenoptera, Proceratiinae) in Fiji

**DOI:** 10.3897/zookeys.475.8761

**Published:** 2015-01-22

**Authors:** Francisco Hita Garcia, Eli M. Sarnat, Evan P. Economo

**Affiliations:** 1 Entomology, California Academy of Sciences, San Francisco, U.S.A.; 2 Okinawa Institute of Science and Technology Graduate University, Onna-son, Okinawa, 904-0495, Japan; 3 University of Illinois Department of Entomology, Illinois, USA

**Keywords:** Endemic, identification key, Melanesia, Oceania, *Proceratium silaceum* clade, South Pacific, taxonomy

## Abstract

The Fiji archipelago harbours a surprisingly diverse and endemic ant fauna, despite its isolated and remote location in the South Pacific. The ant genus *Proceratium* is present on Fiji with three endemic species, of which *Proceratium oceanicum* De Andrade, 2003 and *Proceratium relictum* Mann, 1921 were previously known. In this study we describe the third species: *Proceratium vinaka* **sp. n.** All three species are members of the widespread and species-rich *Proceratium silaceum* clade. In order to integrate the new species into the current taxonomic system, we present an illustrated identification key to the worker caste of the three Fijian species. In addition, we provide a detailed description of *Proceratium vinaka*, as well as species accounts for the other two species, which include diagnoses, taxonomic discussions, specimen photographs, and a distribution map.

## Introduction

The ant genus *Proceratium* Roger, 1863 is distributed throughout all zoogeographical regions, and contains 81 extant and 5 fossil species (Baroni Urbani and de Andrade 2003; Bolton 2014). Despite this global distribution, most *Proceratium* species are comparatively rare and seldom collected, which is likely due to their cryptobiotic lifestyle (Baroni Urbani and de Andrade 2003). In addition, the natural history of this genus remains mostly unknown, and is only available from a few fragmentary reports based on a small number of the known species. It seems that *Proceratium*, like the closely related *Discothyrea* Roger, are specialised predators of arthropod eggs. Brown repeatedly reported several species carrying, storing, and feeding on spider eggs (1958a, 1974, 1980). More recently, Fisher (2005) also observed the same behaviour and diet in *Proceratium avium* Brown, 1974 from Mauritius. Interestingly, there is also evidence that in a few species of *Proceratium* the queens practice occasional larval hemolymph feeding (Masuko 1986; Baroni Urbani and De Andrade 2003). Most species seem to nest in the soil, below leaf litter, in rotten wood, under stones, or more rarely in tree branches (Brown 1958a, 1974; Baroni Urbani and de Andrade 2003; Fisher 2005). Colonies of *Proceratium* were traditionally considered to be relatively small, mostly containing much fewer than 100 workers (Brown 1958a, 1958b; Leston 1971), but recent studies show that they can have a few hundred workers in some species (Onoyama and Yoshimura 2002; Fisher 2005). Fisher (2005) reported the largest colony so far encountered with ca. 350 workers for *Proceratium avium*.

The taxonomy of the genus is in a moderately good condition. Baroni Urbani and de Andrade (2003) revised the genus on a global scale and provided a morphology-based phylogeny. They also divided the genus into several mutually exclusive clades and provided identification keys to all regions. Nevertheless, due to the rarity of collections and specimens available in 2003, the data about intra- and interspecific variation is sometimes limited and will very likely have to be modified in the future when more material becomes available. A few species have been discovered and described since 2003 (Fisher 2005; Xu 2006; Hita Garcia et al. 2014) and more species can be expected in the future. Baroni Urbani and De Andrade (2003) recognised the following eight species from Oceania and provided an identification to the worker caste: *Proceratium austronesicum* De Andrade, 2003 (Papua New Guinea), *Proceratium caledonicum* De Andrade, 2003 (New Caledonia), *Proceratium ivimka* De Andrade, 2003 (Papua New Guinea), *Proceratium oceanicum* De Andrade, 2003 (Fiji), *Proceratium papuanum* Emery, 1897 (Malaysia, Indonesia, Papua New Guinea, Philippines, Solomon Islands), *Proceratium politum* De Andrade, 2003 (New Caledonia), *Proceratium relictum* Mann, 1921 (Fiji), and *Proceratium snellingi* Baroni Urbani & De Andrade, 2003 (Papua New Guinea). All eight species belong to the *Proceratium silaceum* clade sensu Baroni Urbani and De Andrade (2003), which is the most species-rich clade within the genus with more than 30 described species distributed throughout most zoogeographic regions.

Here, we revise the genus *Proceratium* in Fiji and describe *Proceratium vinaka* **sp. n.**, which was presented as the morphospecies *Proceratium* sp. FJ01 in Sarnat and Economo (2012). We place the new species in the *Proceratium silaceum* clade, which increases the known *Proceratium* diversity in Oceania to nine species. *Proceratium vinaka* is the third *Proceratium* species from Fiji, and we provide an illustrated identification key to the three Fijian species. In addition to the detailed species description of the new species, we give species accounts for *Proceratium oceanicum* and *Proceratium relictum*. We also present diagnoses, high-quality specimen photographs, and distribution maps for the Fijian species.

## Abbreviations of depositories

The collection abbreviations mostly follow Evenhuis (2014). The material upon which this study is based is located and/or was examined at the following institutions:

MCZC Museum of Comparative Zoology, Harvard University, Cambridge, U.S.A.

USNM United States National Museum of Natural History, Washington D.C., U.S.A.

BPBM Bernice Pauahi Bishop Museum, Honolulu, U.S.A.

## Material and methods

The measurements were taken with a Leica M165 C stereomicroscope equipped with an orthogonal pair of micrometres at a magnification of 100×. Measurements are presented in mm to two decimal places. The measurements and indices used in this study are based on Ward (1988), Snelling and Cover (1992), Baroni Urbani and de Andrade (2003), and Hita Garcia et al. (2014), with the exception of PeL (see note below):

EL Eye length: maximum length of eye measured in oblique lateral view.

HL Head length: maximum measurable distance from the mid-point of the anterior clypeal margin to the mid-point of the posterior margin of head, measured in full-face view. Impressions on anterior clypeal margin and posterior head margin reduce head length.

HLM Head length with mandibles: maximum head length in full-face view including closed mandibles.

HW Head width: maximum head width directly behind the eyes, measured in full-face view.

HFeL Hind femur length: maximum length of hind femur measured along its external face.

HTiL Hind tibia length: maximum length of hind tibia measured on its external face.

HBaL Hind basitarsus length: maximum length of hind basitarsus measured along its external face.

LT3 Abdominal tergum III length: maximum length of abdominal tergum III (=length of segment III) in lateral view.

LS4 Abdominal sternum IV length: maximum length of abdominal sternum IV following Ward (1988).

LT4 Abdominal tergum IV length: maximum length of abdominal tergum IV following Ward (1988).

PeL Petiolar length: maximum length of the petiolar node in dorsal view. [Note: we have modified PeL compared to previous publications by measuring only the petiolar dorsum without the anterior prolongation of the petiole. Since the species treated here all possess a squamiform node, we are confident that measuring the dorsum of the node provides more comparative information than measuring the petiole as a whole.]

PeW Petiolar width: maximum width of petiole measured in dorsal view.

SL Scape length: maximum length of scape shaft excluding basal condyle.

TL Total body length: combined length of HLM + WL + PeL + LT3 + LT4.

WL Weber’s length: diagonal length of mesosoma in lateral view from the anterior-most point of pronotal slope (excluding neck) to posteroventral margin of propodeal lamella or lobe.

CI Cephalic index: HW / HL × 100

OI Ocular index: EL / HW × 100

SI Scape index: SL / HL × 100

DPeI Dorsal petiole index: PeW / PeL × 100

ASI Abdominal segment index: LT4 /LT3 × 100

IGR Gastral reflexion index: LS4 / LT4

The morphological terminology used in this study follows Snelling and Cover (1992) and Baroni Urbani and de Andrade (2003) with a few important modifications outlined in Hita Garcia et al. (2014). The use of postpetiole, gastral segments and abdominal segments in Baroni Urbani and de Andrade (2003) is confusing at times. To avoid this we do not use the terms postpetiole and gaster and instead use abdominal segment III for the postpetiole and abdominal segment IV for the gastral segment I following Fisher (2005). In addition, instead of the term ‘spur of foretibia’ used by Baroni Urbani and de Andrade (2003), we prefer the term ‘calcar of strigil’ following Keller (2011). Furthermore, in order to adequately describe pubescence and pilosity we follow Wilson (1955) and use the terms ‘erect’, ‘suberect’, ‘subdecumbent’, ‘decumbent’ and ‘appressed’. The terminology for the description of surface sculpturing is based on Harris (1979).

This description of *Proceratium vinaka* and its separation from *Proceratium oceanicum* and *Proceratium relictum* is based on the worker caste. However, the thorough ant inventory performed on the archipelago also yielded many male specimens (Sarnat and Economo 2012). All male specimens were captured from malaise traps, and thus cannot be reliably associated with the worker castes of their respective species in lieu of molecular techniques or future collection of nest series. Although preliminary morphological analysis suggests the male-worker caste associations proposed in Sarnat and Economo (2012) are likely valid, these hypotheses require additional testing before the males can confidently be described as conspecific with the nominal Fijian *Proceratium*. To this end we are including specimens of all available castes in an on-going molecular phylogenetic study, the results of which will be presented elsewhere. We refrain from including males in the distribution maps of the current study, but acknowledge that the ranges of the three species treated here are likely much broader than those illustrated in Figure 6.

## Results

### Synopsis of Fiji *Proceratium* species

*Proceratium oceanicum* De Andrade, 2003

*Proceratium relictum* Mann, 1921

*Proceratium vinaka* Hita Garcia, Sarnat & Economo, sp. n.

### Identification key to Fiji *Proceratium* species (workers)

**Table:** 

1	Significantly smaller species (HW 0.52; WL 0.66); lateral expansions of frontal carinae conspicuously triangular and acute (Fig. 1A); petiolar node moderately squamiform (DPeI 263), and only weakly narrowing from base to apex; subpetiolar process rounded, not dentiform nor spiniform (Fig. 1D)	***Proceratium vinaka***
–	Significantly larger species (HW 0.76–1.03; WL 0.99–1.38); lateral expansions of frontal carinae weakly triangular and moderately rounded (Fig. 1B, C); petiolar node extremely squamiform (DPeI 620–693) and strongly narrowing from base to apex; subpetiolar process conspicuously dentiform or spiniform (Fig. 1E, F)	**2**
2	Smaller species (HW < 0.80; WL < 1.10); in full-face view head thinner (CI 93); in profile propodeum rounded from dorsum to declivity (Fig. 2A); subpetiolar process thinly dentiform (Fig. 1E)	***Proceratium oceanicum***
–	Larger species (HW > 1.00; WL > 1.30); in full-face view approximately as long as wide (CI 101); in profile propodeum with weak angles from dorsum to declivity (Fig. 2B); subpetiolar process thickly spiniform (Fig. 1F)	***Proceratium relictum***

### Notes on the *Proceratium* fauna of Fiji

In this study we describe a new species collected during a recent survey of the ant fauna of Fiji (Sarnat and Economo 2012). Like the majority of the Fijian ant fauna, the new species is endemic to the archipelago. This high endemism is due in part to several *in situ* ant radiations, some of which (e.g. *Lordomyrma* Emery, *Pheidole* Westwood) have been confirmed with molecular analyses (Lucky and Sarnat 2010; Sarnat and Moreau 2011) while others (e.g. *Camponotus* Mayr, *Strumigenys* Smith, *Leptogenys* Roger) are suspected based on morphology (Sarnat and Economo 2012). The ecological and evolutionary assembly processes involved in the development of these faunas have also been the subjects of considerable historical (Wilson 1961) and contemporary (Economo and Sarnat 2012) interest.

The Fiji archipelago harbours an interesting assemblage of endemic *Proceratium* species. *Proceratium oceanicum* and *Proceratium relictum* are certainly the most unusual members of the *Proceratium silaceum* clade, as already pointed out by Baroni Urbani and De Andrade (2003). The extremely squamiform petiolar node shape is unique within the clade, and is also not seen in any other member of the genus. In addition, the ventral petiolar process is dentiform or spiniform and not rectangular or triangular lamelliform as in most other clade members. Indeed, the shape of the ventral process of *Proceratium oceanicum* and *Proceratium relictum* is closer to the shape observable in most members of the *Proceratium stictum* clade than to all other *Proceratium silaceum* clade species. Consequently, it is possible that *Proceratium oceanicum* and *Proceratium relictum* represent an independent evolutionary lineage outside the widespread *Proceratium silaceum* clade that could have developed their unique morphology due the remote and isolated position of the archipelago. However, due to the fact that all species in Oceania belong to the *Proceratium silaceum* clade, it is also possible that *Proceratium oceanicum* and *Proceratium relictum* constitute a small, but distinct, lineage derived from a basal branch of the *Proceratium silaceum* clade. This latter explanation seems more likely if one compares the shape of the petiolar node. Only the members of the *Proceratium silaceum* clade display a squamiform node, and *Proceratium oceanicum* and *Proceratium relictum* are unique mostly in the extent to which their petiolar nodes narrow apically. Nevertheless, only a comprehensive phylogenetic study combining molecular and morphological data might reveal the evolutionary relationships within the clade and among the other *Proceratium* clades. The newly described *Proceratium vinaka* does not share the aberrant morphology observed in its two Fijian congeners. Rather, it is morphologically similar to the other clade species found on New Caledonia, Papua New Guinea, Solomon Islands, or Australia. It can be well distinguished from all of them by the character combination and diagnosis provided below.

#### Proceratium oceanicum

Taxon classificationAnimaliaHymenopteraFormicidae

De Andrade, 2003

**Figure 1.**
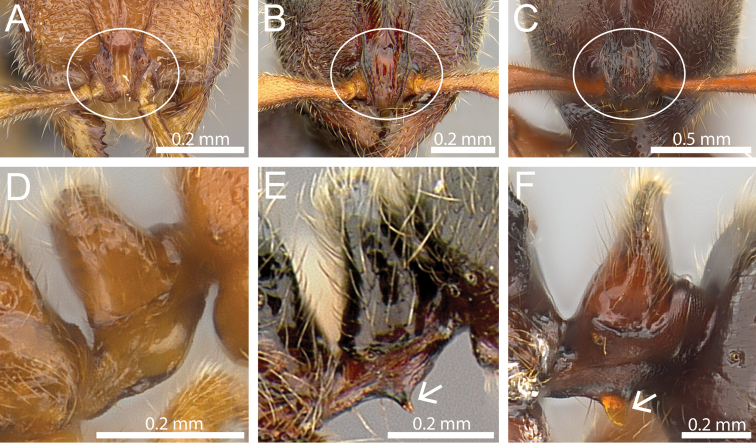
Anterior part of cephalic dorsum in full-face view showing clypeus and frontal carinae (within white ellipse) and petiolar node in profile (white arrows indicate subpetiolar process). **A, D** *Proceratium vinaka* (CASENT0171053) **B, E** *Proceratium oceanicum* (CASENT0171053) **C, F** *Proceratium relictum* (CASENT0194740).

**Figure 2.**
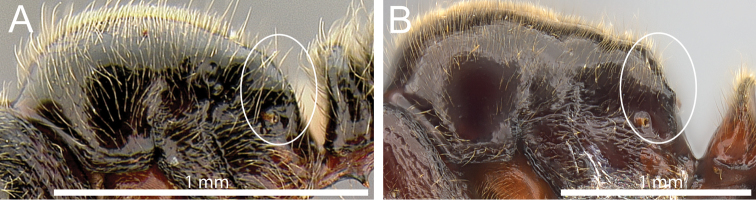
Mesosoma in profile showing posterodorsal propodeum (within white ellipse). **A** *Proceratium oceanicum* (CASENT0171053) **B** *Proceratium relictum* (CASENT0194740).

**Figure 3.**
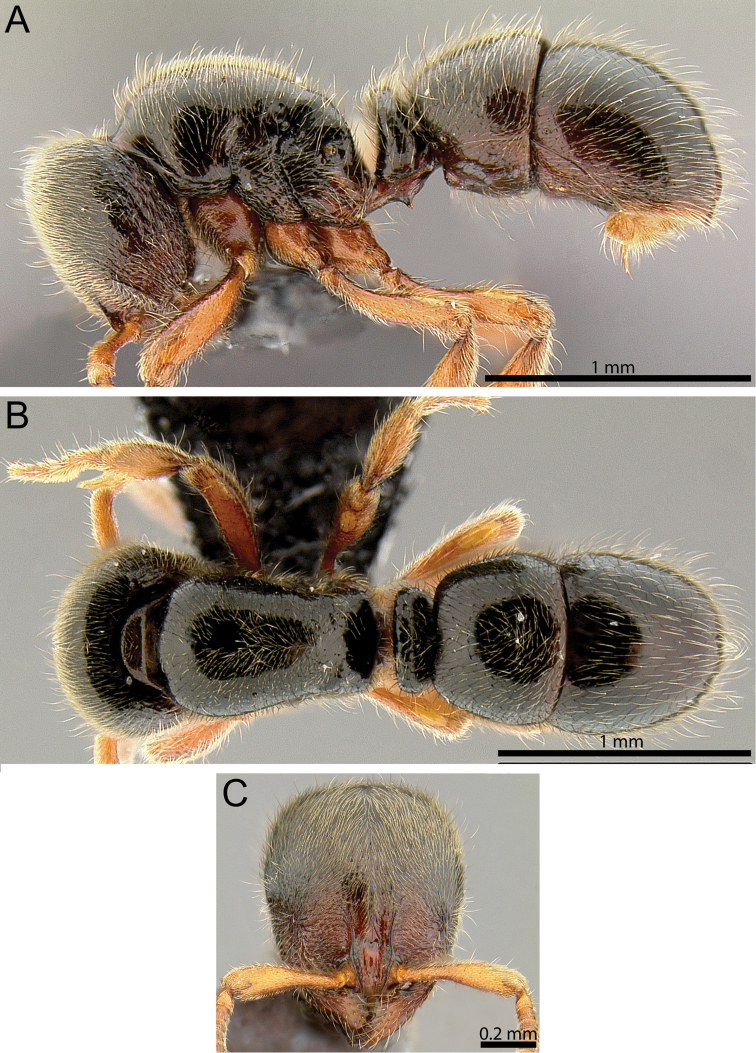
*Proceratium oceanicum* (CASENT0171053). **A** Body in profile **B** Body in dorsal view **C** head in full-face view.

Figs 1B, E , 2A , 3 , 6

Proceratium oceanicum De Andrade, 2003: 310. [see also: Sarnat and Economo 2012: 166]

##### Type material.

**Holotype**, pinned worker, FIJI, Viti Levu, Nadarivatu, -17.5667°, 177.967°, rainforest, on soil, under grass, 16.II.1962, (*R.W. Taylor*) (ANIC: ANIC32-017668) [not examined].

##### Non-type material examined.

FIJI: Taveuni, Mt. Devo, 3.9 km SE Tavuki Village, -16.83278°, -179.97343°, 775 m, ex soil, leaf litter, decaying wood, 16.VI.2005 (*E.M. Sarnat*); Viti Levu, Koroyanitu Eco Park 5.0 km NE Abaca Village, -17.66667°, 177.5525°, 700 m, disturbed forest, sifted litter, 19.IV.–14.V.2003 (*M. Tokotaa*); Viti Levu, Naqaranibuluti Nature Reserve, near summit, 0.75 km SE Nadarivatu, -17.57278°, 177.9725°, 1000 m, primary rainforest, 26.VIII.2006 (*E.M. Sarnat*); Viti Levu, Nausori Highlands, 12.I.1972 (*W.L. Brown*).

##### Diagnosis.

The following character combination distinguishes *Proceratium oceanicum* from the remainder of the *Proceratium silaceum* clade: relatively larger species (HW 0.76–0.78; WL 0.99–1.00); in full-face view head weakly longer than wide (CI 93); lateral expansions of frontal carinae weakly triangular and moderately rounded; petiolar node extremely squamiform (DPeI 680–693) and strongly narrowing from base to apex; subpetiolar process thinly dentiform.

##### Worker measurements

(N=2). TL 3.38–3.41; EL 0.03; SL 0.55–0.58; HL 0.81–0.83; HLM 1.00–1.02; HW 0.76–0.78; WL 0.99–1.00; HFeL 0.63; HTiL 0.48–0.50; HBaL 0.38–0.40; PeL 0.63; PeW 0.43; DPeI 680–693; LT3 0.54–0.56; LS4 0.35–0.40; LT4 0.71–0.78; OI 4; CI 93; SI 68–70; IGR 0.49–0.52; ASI 133–139.

##### Distribution and biology.

Workers of *Proceratium oceanicum*, even though rarely encountered, were sampled from Viti Levu and Taveuni, but tentatively associated males also suggest its presence on Vanua Levu. All collections are from primary or disturbed rainforest. Unfortunately, there is no available data on the biology of *Proceratium oceanicum*.

##### Taxonomic notes.

As already outlined above, *Proceratium oceanicum* and *Proceratium relictum* are highly distinctive species that can be easily distinguished from all other congeners by the extremely squamiform petiolar node. This character, among others, also separates both clearly from the new species *Proceratium vinaka*. Despite its sympatric occurrence, *Proceratium oceanicum* and *Proceratium relictum* are not likely to be confused. The latter is significantly larger in size (HW > 1.00; WL > 1.30), has a noticeably broader head (CI 101), and the subpetiolar process is thickly spiniform, whereas *Proceratium oceanicum* is conspicuously smaller (HW < 0.80; WL < 1.10), possesses a narrower head (CI 93), and its subpetiolar process thinly dentiform. Additionally, in profile the propodeum of *Proceratium oceanicum* is rounded while it is weakly, but clearly marginate in *Proceratium relictum*.

#### Proceratium relictum

Taxon classificationAnimaliaHymenopteraFormicidae

Mann, 1921

**Figure 4.**
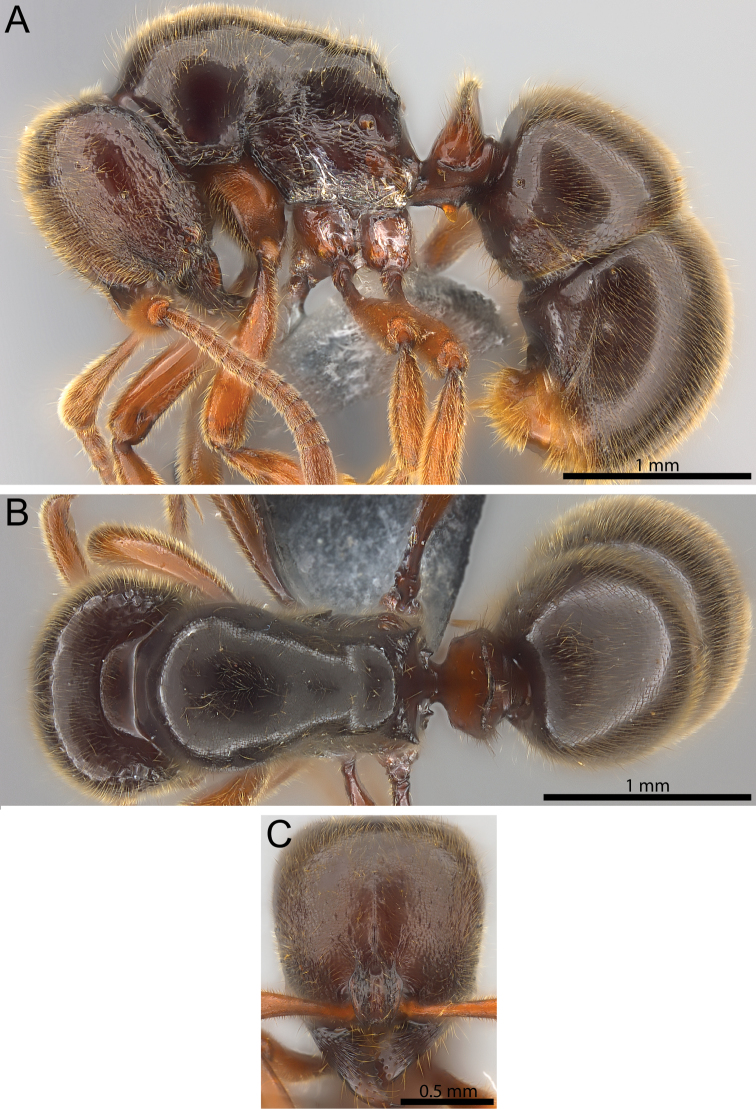
*Proceratium relictum* (CASENT0194740). **A** Body in profile **B** Body in dorsal view **C** head in full-face view.

Figs 1C, F , 2B , 4 , 6

Proceratium relictum Mann, 1921: 413. [see also: Baroni Urbani and de Andrade 2003: 306; Sarnat and Economo 2012: 167]

##### Type material.

**Syntypes**, pinned workers and queen, FIJI, Taveuni, Somosomo (*W.M. Mann*) (MCZC, USNM) [examined].

##### Non-type material examined.

FIJI: Taveuni, Mt. Devo, 3.6 km SE Tavuki Village, -16.83056°, -179.97433°, 734 m, garden/forest edge, on ground, foraging, hand collection, 22.III.2005 (*E.M. Sarnat*); Vanua Levu, Mt. Delaikoro, Delaikoro Rd., 3.6 km SE Dogoru Village, -16.57525°, 179.31638°, 699 m, primary rainforest, 31.VIII.2006 (*M. Tokotaa*); Vanua Levu, 1.5 km N Yasawa Village, -16.46806°, 179.64362°, 300 m, disturbed forest, 1.IX.2003 (*A. Rakabula*).

##### Diagnosis.

The following combination of characters separates *Proceratium relictum* from the remainder of the *Proceratium silaceum* clade: comparatively large species (HW 1.03; WL 1.38); in full-face view head approximately as long as wide (CI 101); lateral expansions of frontal carinae weakly triangular and moderately rounded; petiolar node extremely squamiform (DPeI 620) and strongly narrowing from base to apex; subpetiolar process thickly spiniform.

##### Worker measurements

**(N=1).** TL 4.45; EL 0.04; SL 0.78; HL 1.01; HLM 1.28; HW 1.03; WL 1.38; HFeL 0.98; HTiL 0.71; HBaL 0.67; PeL 0.83; PeW 0.52; DPeI 620; LT3 0.69; LS4 0.42; LT4 1.02; OI 4; CI 101; SI 77; IGR 0.41; ASI 148.

##### Distribution and biology.

*Proceratium relictum* seems to be restricted in its distribution to the two northern islands Taveuni and Vanua Levu. Tentatively associated males collected from malaise traps support this restricted distribution and absence of the species from Viti Levu. The species appears to be tolerant of at least moderate levels of disturbance as it was collected from rainforest, disturbed forest, and forest edge garden. Collections of workers are rare and there is no available information about its natural history.

##### Taxonomic notes.

*Proceratium relictum* and *Proceratium oceanicum* form a very close species pair easily distinguishable from the new species *Proceratium vinaka* and all other *Proceratium* species by the extremely modified petiolar node. Detailed information on how to separate *Proceratium relictum* from *Proceratium oceanicum* is presented in the species account of the latter and the identification key.

#### Proceratium vinaka sp. n.

Taxon classificationAnimaliaHymenopteraFormicidae

http://zoobank.org/A6667E4F-4322-4895-8ECB-C8DA6DDAC48A

Figs 1A, D , 5 , 6

##### Type material.

**Holotype**, pinned worker, FIJI, Viti Levu, Savatu Dist., Mt. Tomanivi 2.4 km E Navai Vlg., -17.61806°, 178.0055°, 950 m, mid-elevation rainforest, soil, leaf litter, decaying wood, collection code EMS#2153-4, 25.VI.2005 (*E.M. Sarnat*) (BPBM: CASENT0187587).

##### Diagnosis.

*Proceratium vinaka* differs from the other members of the *Proceratium silaceum* clade by the following combination of characters: relatively smaller species (HW 0.52; WL 0.66); in full-face view head weakly longer than wide (CI 93); lateral expansions of frontal carinae conspicuously triangular and acute; petiolar node moderately squamiform (DPeI 263) and only weakly narrowing from base to apex; subpetiolar process rounded, not dentiform nor spiniform.

##### Worker measurements

**(N=1).** TL 2.41; EL 0.03; SL 0.37; HL 0.56; HLM 0.79; HW 0.52; WL 0.66; HFeL 0.38; HTiL 0.29; HBaL 0.24; PeL 0.10; PeW 0.25; DPeI 263; LT3 0.37; LS4 0.27; LT4 0.51; OI 6; CI 93; SI 66; IGR 0.54; ASI 138.

##### Worker description.

In full-face view head longer than broad (CI 93), sides weakly convex, gently broadening posteriorly, vertex shallowly concave. Clypeus conspicuously reduced, relatively narrow, and anteriorly truncate. Frontal carinae relatively short, moderately separated, and not covering antennal insertions, approximately parallel on anterior third and strongly diverging posteriorly, lateral expansions of frontal carinae very broad, raised, and conspicuously triangular and acute; frontal area weakly concave; cephalic dorsum medially with weak carina. Eyes reduced, very small (OI 6), consisting of single ommatidium and located on midline of head. Antennae 12-segmented, scapes short (SI 66), not reaching posterior head margin and noticeably thickening apically. Mandibles elongate-triangular; masticatory margin of mandibles with eight teeth/denticles in total, apical tooth long and acute, second tooth from apex smaller and less acute, remaining six denticles significantly smaller and blunt. Mesosoma in profile moderately convex and clearly shorter than maximum head length including mandibles. Lower mesopleurae with well demarcated sutures, no other sutures developed on lateral or dorsal mesosoma; mesopleurae not inflated posteriorly; propodeum in profile unarmed and rounded, propodeal lobes weakly developed, lamellate and blunt; declivitous face of propodeum gently sloping posteriorly; in posterodorsal view sides of propodeum separated from declivitous face by weak margins; in profile propodeal spiracle rounded and above mid height. Legs moderately long; all tibiae with pectinate spur; calcar of strigil without basal spine; pretarsal claws simple; arolia absent. Petiolar node in profile moderately squamiform, high, and subrectangular, anterior face of petiole relatively straight, node weakly narrowing from base to apex, dorsum of node weakly convex; petiole in dorsal view much broader than long and transverse, around 2.6 times broader than long (DPeI 263); ventral process of petiole relatively reduced, inconspicuous, convex, and without any rectangular, dentiform, or spiniform projections. In dorsal view abdominal segment III anteriorly much broader than petiole; its sides diverging posteriorly; abdominal sternite III anteromedially with a marked subtriangular projection appearing convex in profile. Constriction between abdominal segment III and IV conspicuously impressed. Abdominal segment IV moderately recurved (IGR 0.54), conspicuously rounded on its curvature, especially posteriorly; abdominal tergum IV around 1.4 times longer than abdominal segment III (ASI 138); remaining abdominal tergites and sternites relatively inconspicuous and curved ventrally. All dorsal surfaces of body (including antennal scapes and legs) covered with dense mat of relatively short, decumbent to erect hairs combined with fewer, but significantly longer, erect hairs.

Mandibles conspicuously striate at the base and mostly smooth and shining towards apex; sides of head and anterior cephalic dorsum irregularly foveolate and/or punctate and irregularly rugulose, sculpture on posterior of cephalic dorsum very weak and shining; sculpture on mesosoma, petiole, abdominal tergites III and IV weakly to moderately irregularly foveolate and/or punctate, generally appearing quite smooth and shiny, abdominal sternites III and IV irregularly foveolate and/or punctate and irregularly rugulose, rough in appearance. Body colour uniformly yellowish to light orange brown.

##### Etymology.

The name of new species is Fijian and means ‘thank you’ or ‘hello’. With this we want to dedicate the new species to the people of Fiji for their hospitality and kindness shown to EMS and EPE during their years of fieldwork on the archipelago. The species epithet is a nominative noun in apposition, and thus invariant.

##### Distribution and ecology.

The single known worker of *Proceratium vinaka* was collected at Mt. Tomanivi on Viti Levu. The type locality is a relatively pristine mid-elevation rainforest. Nevertheless, several tentatively associated males from malaise traps suggest that *Proceratium vinaka* has a much broader distribution and is also found on Taveuni and Vanua Levu. As in the cases of *Proceratium oceanicum* and *Proceratium relictum*, there is no information on the biology of the new species.

##### Taxonomic notes.

Despite the morphological similarity of most species of the *Proceratium silaceum* clade, *Proceratium vinaka* possesses an interesting character combination that renders it easily identifiable within the *Proceratium* fauna of Oceania. It cannot be confused with the other two *Proceratium* species found on Fiji. Both, *Proceratium oceanicum* and *Proceratium relictum*, have extremely squamiform petiolar nodes that strongly narrow from base to apex, whereas *Proceratium vinaka* has a moderately squamiform node that narrows only very weakly from base to apex. This node shape is characteristic for the *Proceratium silaceum* clade and found in all species except *Proceratium oceanicum* and *Proceratium relictum*. In addition, the latter two species have either a dentiform or spiniform ventral petiolar process, which contrasts with the very much reduced and convex process of *Proceratium vinaka*. Interestingly, this highly reduced ventral process seen in *Proceratium vinaka* is quite unique and not found in any other member of the *Proceratium silaceum* clade in Oceania. All other species have either a well-developed lamelliform and approximately rectangular process, or the process is dentiform or spiniform. Another character that distinguishes *Proceratium vinaka* from *Proceratium oceanicum* and *Proceratium relictum* is the development of the lateral expansions of the frontal carinae, which are weakly triangular and moderately rounded in the latter two species, whereas they are conspicuously triangular and acute in *Proceratium vinaka*. This also separates it from other morphologically similar species found in Oceania, such as *Proceratium caledonicum*, *Proceratium papuanum*, or *Proceratium politum* since they all have rounded or subtriangular extensions that are never as acute as in *Proceratium vinaka*.

*Proceratium vinaka* was treated as *Proceratium* sp. FJ01 in Sarnat and Economo (2012).

**Figure 5.**
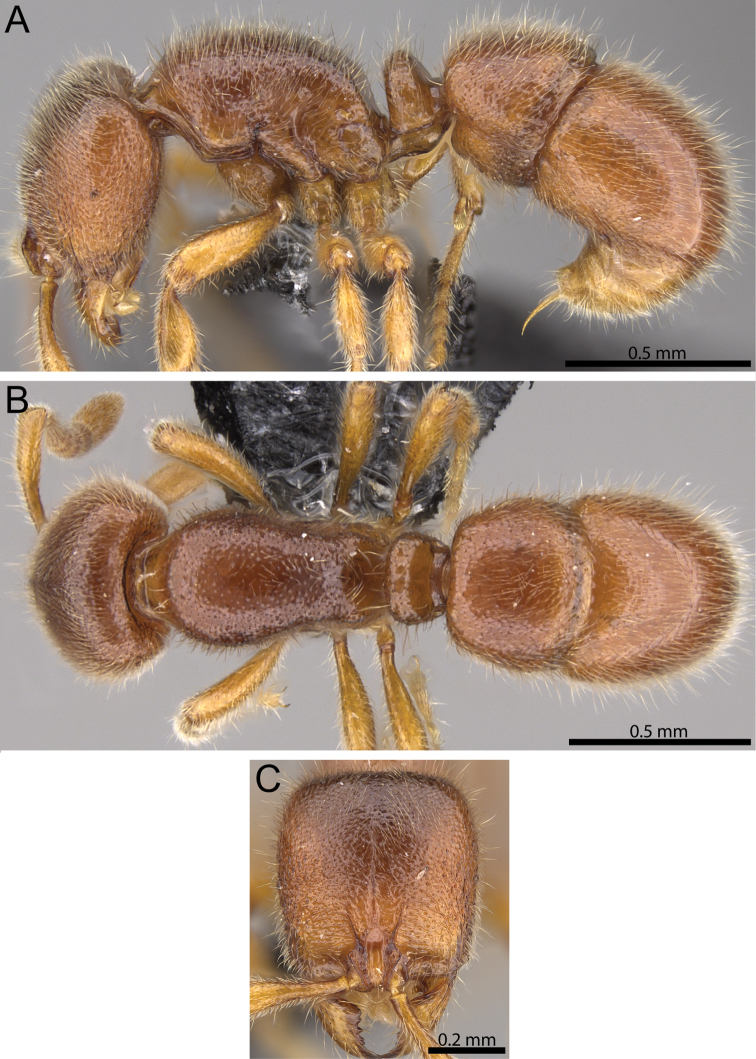
*Proceratium vinaka* (CASENT0187587). **A** Body in profile **B** Body in dorsal view **C** Head in full-face view.

**Figure 6.**
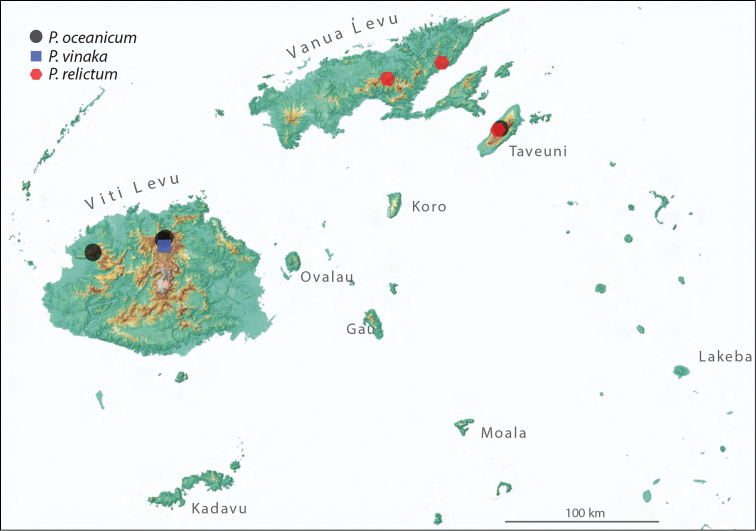
Map of Fiji showing the known distribution of the three species of *Proceratium* known from the archipelago (*Proceratium oceanicum* – black circle; *Proceratium relictum* – red hexagon; *Proceratium vinaka* – blue square).

## Supplementary Material

XML Treatment for Proceratium oceanicum

XML Treatment for Proceratium relictum

XML Treatment for Proceratium vinaka
